# Anesthetic Management of Emergency Insulinoma Resection: Case Report and Review of Literature

**DOI:** 10.7759/cureus.49425

**Published:** 2023-11-26

**Authors:** Noor ul Huda, Muhammad H Chatha, Sheharyar Baig, Ahsun Khan, Ahmed Bilal Akhtar

**Affiliations:** 1 Anesthesia, Shaukat Khanum Memorial Cancer Hospital and Research Centre, Lahore, PAK; 2 Anesthesia and Pain Management, Shaukat Khanum Memorial Cancer Hospital and Research Centre, Lahore, PAK; 3 Anesthesia and Critical Care, Shaukat Khanum Memorial Cancer Hospital and Research Centre, Lahore, PAK; 4 Anesthesia and Critical Care, King Faisal Specialist Hospital and Research Centre, Riyadh, SAU

**Keywords:** anesthesia, hypoglycemia, case report, anaesthetic, insulinoma

## Abstract

Insulinoma, a neuroendocrine tumor originating from pancreatic islets, presents unique challenges in diagnosis and management. We present a case of a 73-year-old female with recurrent hypoglycemia leading to syncope, who underwent emergency pancreatectomy for a secreting insulinoma with multiple comorbidities. This case report aims to shed light on the complexities of insulinoma management and the importance of tailored perioperative strategies. The patient, presenting with severe hypoglycemia, was admitted for optimization. Preoperative assessment labeled her as ASA IVE and indicated a high risk of perioperative morbidity. General anesthesia, invasive monitoring, and epidural anesthesia were planned. Intraoperative glucose control was crucial, achieved with continuous blood glucose monitoring, octreotide administration, and insulin titration. The patient was extubated post-surgery, and pain was managed with epidural infusion. She was discharged on the 4th postoperative day with follow-up care.

Insulinoma diagnosis relies on clinical, biochemical, and imaging tests, with 72-hour fasting as the gold standard. Localizing the tumor within the pancreas is essential for surgical success, often requiring invasive techniques. Surgical resection remains the definitive treatment, while medical management may be necessary in select cases. Anesthetic management should prioritize agents that minimize the cerebral metabolic rate for oxygen. Careful intraoperative glucose control and vigilant postoperative monitoring are essential. This case report highlights the intricate management of insulinoma, emphasizing tailored perioperative strategies that balance glucose regulation, anesthesia techniques, and postoperative care. However, the limited existing literature underscores the need for further research to refine anesthesia protocols, glucose control methods, and postoperative care, ultimately improving outcomes for patients with insulinoma.

## Introduction

Insulinoma is a neuroendocrine tumor derived from the pancreatic islets of Langerhans cells. They are the most common type of functional neuroendocrine tumor (NET); however, NET is rare. In the nineteenth century, it was not until the start of the use of insulin for treating diabetes mellitus that insulinoma or the term hyperinsulinism was even in the differential diagnosis for the possible manifestation of hypoglycemia [1].

Sporadically occurring insulinomas are generally benign and singular; however, when linked to multiple endocrine neoplasia type 1 (MEN1), which occurs in approximately 10% of cases, the prognosis is severe. This is due to the potential for malignancy and associated health risks of overproduction of hormones [2].

Patients with insulinoma often present with hypoglycemia, particularly during fasting. However, some individuals may experience decreased blood sugar levels even after eating [3]. Patients with recurrent episodes of hypoglycemia may experience a range of symptoms, including sympathoadrenal and neuroglycopenic symptoms [4]. Additionally, 20-40% of patients may gain weight as a result of overeating to manage hypoglycemia. "Whipple's triad" of fasting or exercise-induced hypoglycemia, plasma glucose less than 50 mg/dL, and symptom relief with glucose delivery serve as the basis for clinical diagnosis [2]. 

Treatment comprises both medical and surgical procedures. The mainstay of treatment is surgical, mostly enucleation, but may require partial or distal pancreatectomy in cases of malignant tumors or if they are in close proximity to the duct. Medical care is primarily aimed at patients awaiting surgery or those who are not candidates for surgery. This includes eating frequent small meals throughout the day to avoid symptoms of hypoglycemia and drug therapy with benzothiazides, somatostatin analogs, verapamil, and phenytoin [5]. We report the anesthetic management of a 73-year-old female undergoing pancreatectomy for a secreting insulinoma with multiple comorbidities.

## Case presentation

**Figure 1:**
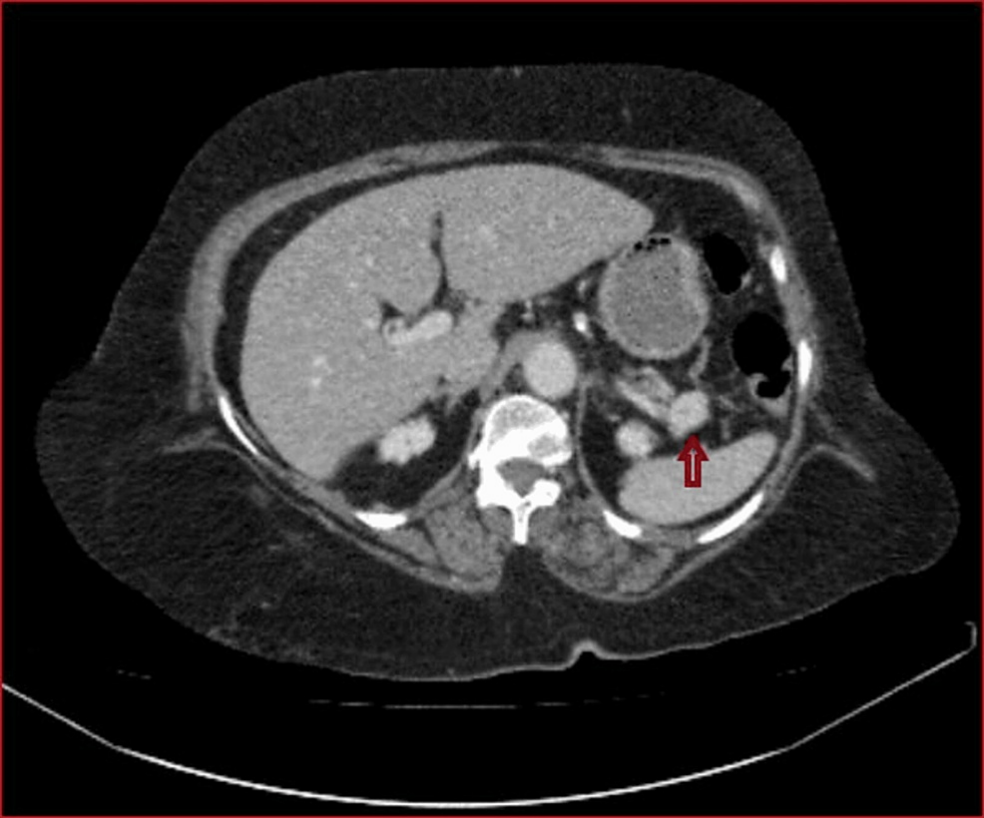
Well-defined 1.7 cm arterially enhancing lesion in the tail of pancreas (shown by red arrow)

A 73-year-old female (height 143 cm, weight 76 kg) presented with a 1-year history of irritation, dizziness, and recurrent hypoglycemia often leading to syncope. Her family history was negative for malignancy. An initial workup was done in which her computed tomography (CT) abdomen with contrast showed a mass in the tail of pancreas (Figure 1). Tissue diagnosis was not available at that time. The blood tests on the initial presentation are shown in Table 1. The patient was a known case of hypertension, (controlled on medication) and was obese with a body mass index of 37 kg/m2. She had a moderate to high risk for postoperative obstructive sleep apnea (as per the STOP-Bang score of 5/8, Annexure 1). The patient had limited functional capacity owing to severe osteoarthritis in bilateral knee joints. The patient was already taking prednisolone for 1 week.

**Table 1:** The blood tests on initial presentation HGB: hemoglobin; HCT: hematocrit; MCV: mean corpuscular volume; MCH: mean corpuscular hemoglobin; MCHC: mean corpuscular hemoglobin concentration; PLT: platelet; INR: international normalized ratio; eGFR: estimated glomerular filtration rate; LFTs: liver funtion tests; ALT: alanine aminotransferase; AST: serum aspartate aminotransferase; GGT: gamma-glutamyl transferase; A/G ratio: albumin/globulin ratio

Test	Results/Units	Normal Range
Complete Blood Count		
WBC	9.95x 10^3^/µl	4-10
RBC	5.01 x 10^6 ^/µl	3.8 - 4.8
HGB	12.9 g/dL	12 - 15
HCT	40.6 %	36 - 46
MCV	81 fL	76 - 96
MCH	25.7 pg	27 - 32
MCHC	31.8 g/dL	31.5-34.5
PLT	337 x10.e 3/µl	150-450
Coagulation Profile		
Prothrombin Time	10.6 seconds	9-14
INR (Calculated Value)	0.94	
Urea and Electrolytes		
Sodium	142 mmol/L	133 – 145
Potassium	5.17 mmol/L	3.3 - 5.1
Chloride	108 mmol/L	95 – 108
Urea Nitrogen	26.35 mg/dL	6 – 20
Creatinine	1.14 mg/dL	0.50 - 0.90
eGFR	46.62 mL/min/1.73 m²	>60
Bicarbonate	23.3 mmol/L	22 – 29
LFTs		
ALT	37 U/L	10 – 35
AST	34 U/L	10 – 35
GGT	48 U/L	5 – 36
A/G Ratio	1.03	
Total Bilirubin	0.43 mg/dL	Up to 1.0
Alkaline Phosphatase	122 U/L	35 - 104
Albumin	3.56 g/dL	3.5 - 5.5
Globulin	3.4 g/dL	2.0 - 3.5
Total Protein	7 g/dL	5.5 - 8.0
Glucose Random	28 mg/dL	70-140
C-Peptide	9.21 ng/mL	0.9-7.1
Serum Insulin	12 µIU/mL	Up to 29.1

The patient was booked with the endocrinology team as an outpatient for further management. While awaiting her appointment the patient started having frequent hypoglycemic episodes at home leading to an altered state of consciousness. She presented to the emergency room with severe hypoglycemia of <40mg/dl and drowsiness. She was managed with multiple boluses of 25% dextrose water. In the emergency room, she continued to have recurrent episodes of hypoglycemia leading to neuroglycopenic symptoms including irritability, dizziness, and loss of consciousness. She was started on an intravenous infusion of 5% dextrose saline. The goal was to keep blood sugar level (BSL) between 150-250 mg/dl with titrated dextrose saline infusion and intermittent boluses of 25% dextrose water. Her blood sugar level remained low despite all measures.

A surgical consult was sought and after assessment, the surgery team decided to proceed on an emergency basis with the excision of insulinoma. The patient was admitted to the surgical floor for optimization. A preoperative anesthesia assessment was done. She was labeled using the American Society of Anesthesiologists (ASA) score as ASA IVE because of severe hypoglycemia which was a constant threat to life (Annexure 2). Her arterial blood gas analysis showed metabolic acidosis (Table 2). It was explained to the patient that the procedure would carry a risk of 50% perioperative morbidity and 4% mortality according to Portsmouth-physiological and operative severity score for the enumeration of mortality and morbidity (P-POSSUM) scoring (Table 3) (Annexure 3).

**Table 2:** Preoperative versus postoperative comparison of arterial blood gases PCO2: partial pressure of carbon dioxide; PO2: partial pressure of oxygen; HCO3: bicarbonate

Test	Units	Result Preoperative	Result Before Extubation	Normal Range
pH		7.26	7.36	7.35-7.45
PCO2	mmHg	46	32	35-45
PO2	mmHg	95	318	83-108
HCO3	mmol/L	20.6	18.3	21-28
Base Excess	mmol/L	-6.3	-6.7	(-2) - (+3)

**Table 3:** P-POSSUM scoring P-POSSUM: Portsmouth physiological and operative severity score for the enumeration of mortality and morbidity

Physiology Score	Operative Severity Score	Morbidity (%)	Mortality (%)
23	13	56.0	4.1

A tentative plan was made for general anesthesia, invasive monitoring, and epidural anesthesia. The risk of possible postoperative mechanical ventilation or bilevel-positive airway pressure (BiPAP) for obstructive sleep apnea was explained to the patient. She was given incentive spirometry and was encouraged to do it hourly. A written consent was obtained.

The patient was taken to the operating room the next morning for laparoscopic distal pancreatectomy. Sign-in was done as per the WHO checklist. A thoracic epidural catheter was sited at T7-T8 by the consultant anesthetist after attaching standard ASA monitoring. Induction was done with nalbuphine 3 mg, propofol 100 mg, and atracurium 40 mg. The definite airway was secured with endotracheal tube 7.0 using McGrath blade 3 (laryngoscopy grade 2). An arterial line was inserted in the left radial artery. Central venous catheter 7.0 Fr was placed in the right internal jugular vein using ultrasound. Continuous ASA monitoring was recorded on the anesthesia sheet.

**Figure 2:**
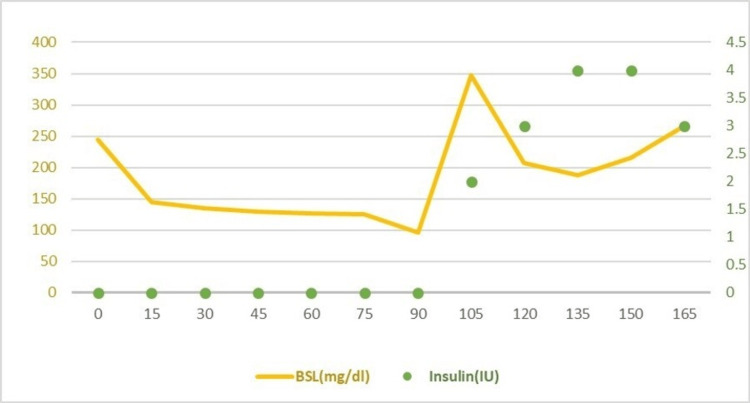
Regular insulin infusion titrated according to BSL readings x-axis: time (minutes) Left y-axis: BSL (mg/dl) Right y-axis: insulin given (IU) BSL: blood sugar level

After induction of general anesthesia intravenous octreotide 100 mcg was given and blood sugar levels were monitored every 15 minutes using On-Call Plus EZ II blood glucometer. Arterial blood gases were checked hourly and remained within acceptable range (Table 3). Ten percent dextrose saline was used as maintenance fluid till the mass was resected. The BSL was maintained between 150-200 mg/dl and no additional boluses of 25% dextrose water were required. After the excision of the mass, BSL started to rise above 300 mg/dl and the patient was started on regular insulin infusion titrated according to BSL readings (Figure 2). The total duration of surgery was 5 hours, with a blood loss of 100 ml. Urine output remained adequate throughout. The course of anesthesia was uneventful.

After the procedure, the patient was extubated in the operating room when fully awake. In the Post-anesthesia Care Unit, her BSL was monitored hourly and she was started on variable rate intravenous insulin infusion (VRIII) protocol in liaison with the endocrinology team. Postoperative pain was managed with an epidural infusion of bupivacaine 0.125% at 10 ml per hour. The patient was monitored in the High Dependency Unit overnight and was stepped down to the surgical floor the next day. Her BSL continued to remain above 250 mg/dl and the endocrinologist advised to start her on glargine 6IU 24 hourly. On 4th postoperative day, she was discharged from the hospital uneventfully and was advised to follow up with the endocrinology team.

## Discussion

By detailing the successful anesthesia management of a 73-year-old female undergoing an emergency pancreatectomy for a secreting insulinoma, our report underscores the significance of tailored perioperative strategies. The delicate balance between glucose regulation, anesthesia techniques, and vigilant postoperative monitoring played a pivotal role in achieving favorable outcomes in this case.

Insulinoma is a rare neuroendocrine tumor that presents a unique set of challenges in diagnosis and management. The incidence of insulinoma is approximately 1-4 per million population per year, with a median age of presentation around 47 years and a mild female preponderance [6].

While reviewing existing literature, we explore the knowledge and insights from the available literature on the perioperative management of insulinoma and other endocrine emergencies. Based on the limited available literature, the anesthetic management of insulinoma remains an area of relative paucity. While the comprehensive review by Peramunage and Nikravan (2020) provides valuable insights into the management of endocrine emergencies, including insulinomas, it highlights the need for further research and in-depth investigations specifically focused on insulinoma perioperative care [7]. The reviews by Cortes et al. (1991) and Suffecool SL (1980) serve as indicators of the limited extent of available literature on this subject [8,9].

Due to the scarcity of literature, the anesthetic management of insulinoma demands greater attention and research efforts. Future studies and evidence-based guidelines are crucial to optimize patient outcomes during insulinoma resection and to address the challenges posed by this rare endocrine tumor.

Diagnosing insulinoma involves a combination of clinical, biochemical, and imaging tests. The 72-hour fasting test is considered the gold standard for diagnosis. During this test, the patient is supervised while fasting, and blood glucose levels are measured regularly. Insulinoma is confirmed if specific criteria, such as blood glucose less than 50 mg/dL with hypoglycemic symptoms, elevated C-peptide levels, and increased serum insulin levels, are met [6,10,11].

Localizing insulinoma within the pancreas is crucial for successful surgical management. Non-invasive imaging modalities like ultrasound, CT, and MRI have limited success rates, while invasive techniques like transhepatic portal venous sampling (THPVS) and intraarterial calcium stimulation test or arterial stimulation and venous sampling (ASVS) have shown better results. Intraoperative ultrasound (IOUS) is particularly useful for localizing small and non-palpable tumors [10,12].

Surgical excision is the definitive treatment for insulinoma, and laparoscopic resection has become the preferred approach. Preoperative medical management may be necessary for patients who are not surgical candidates or waiting for surgery. Medications like diazoxide and somatostatin analogs are used to prevent hypoglycemic episodes. Perioperative glucose management is crucial to prevent hypoglycemia during surgery and control rebound hyperglycemia post-resection [10,13,14].

Anesthetic management during insulinoma surgery is an important consideration. Anesthetic agents that reduce cerebral metabolic rate for oxygen (CMRO2) are preferred. Propofol is recommended over thiopentone sodium due to its more stable effect on glucose regulation. Enflurane and halothane, on the other hand, are not suitable due to their inhibitory effect on pancreatic insulin release and increased sensitivity to insulin [15-17]. Careful intraoperative glucose monitoring and perioperative glucose management are crucial to prevent hypoglycemia during surgery and achieve successful tumor resection. After surgery, the blood glucose level may rise to around 180-230 mg/dL, which may require small doses of insulin and frequent glucose monitoring.

Moreover, recent studies have shed light on the etiology and molecular mechanisms underlying insulinoma. The gene causing multiple endocrine neoplasia type 1 (MEN-1) syndrome, which is associated with 16% of insulinoma cases, is localized in band 11q13 and encodes a protein called menin. The mutations of the MEN1 gene play a significant role in the pathogenesis of sporadic insulinomas. However, other genetic factors may also contribute to the development of insulinomas in non-MEN1 cases [6,18].

## Conclusions

Our case report and review shed light on the intricate management of insulinomas, and neuroendocrine tumors arising from pancreatic islets. The multifaceted nature of these tumors necessitates a comprehensive approach to diagnosis and perioperative care. Insulinoma presents unique challenges in diagnosis and perioperative management. Surgical resection is pivotal, requiring careful glucose control, anesthesia techniques, and postoperative care.

However, the limited existing literature on insulinoma anesthesia management emphasizes the need for continued research. Future studies can delve deeper into optimal anesthesia protocols, glucose control methodologies, and refined postoperative care. By addressing these gaps, we can enhance our ability to provide safe and effective treatment to patients with insulinoma.

## Appendices

Annexure 1 

**Table 4:** STOP-Bang score STOP-Bang: snoring, tiredness, observed apnea, blood pressure, body mass index, age, neck size, gender; OSA: obstructive sleep apnea

Snoring	Do you snore loudly enough to disrupt a bed partner or be heard through a door?
Tiredness	Are you often noticeably sleepy or fatigued during the day?
Observed Apneas	Has someone seen you gasp, choke, or stop breathing while sleeping?
Pressure	Do you have high blood pressure?
Body mass index (BMI)	BMI > 35
Age	Age above 50
Neck circumference	Having a larger neck (> 16 cm) is a risk factor for OSA.
Gender (sex)	People of male sex have a higher risk of OSA
STOP-Bang Score	Risk of OSA
0 to 2	Low risk of obstructive sleep apnea
3 to 4	Intermediate risk of obstructive sleep apnea
5 to 8	High risk of obstructive sleep apnea

Annexure 2

The American Society of Anesthesiologists (ASA) score is a subjective assessment of a patient’s overall health that is based on five classes (I to V).

Class I: The patient is a completely healthy fit patient.

Class II: The patient has mild systemic disease.

Class III: The patient has a severe systemic disease that is not incapacitating.

Class IV: The patient has an incapacitating disease that is a constant threat to life.

Class V: A moribund patient who is not expected to live 24 hours with or without surgery.

E. Emergency surgery.

Annexure 3

**Table 5:** POSSUM physiological score POSSUM: physiological and operative severity score for the enumeration of mortality and morbidity; WCC: white cell count

Physiological score

Variable	1 point	2 points	4 points	8 points
Age	≤ 60	61-70	≥ 71	
Cardiac signs chest X-ray	Normal	Cardiac signs or steroids	Edema, warfarin, or borderline cardiomegaly	Raised jugular venous pressure or cardiomegaly
Respiratory signs chest X-ray	Normal	Shortness of breath on exertion mild chronic obstructive airway disease	Shortness of breath on stairs, Moderate chronic obstructive disease	Shortness of breath at rest, Any other change
Systolic blood pressure (mmHg)	110-130	131-170 or 100-109	≥ 171 or 90-99	≤ 89
Pulse (beats/min)	50-80	81-100 or 40-49	101-120	≥ 121 or ≤ 39
Glasgow coma score	15	12-14	9-11	≤ 8
Urea nitrogen (mmol/L)	< 7.5	7.6-10	10.1-15	≥15.1
Sodium (mEq/L)	> 136	131-135	126-130	≤ 125
Potassium (mEq/L)	3.5-5	3.2-3.4 or 5.1-5.3	2.9-3.1 or 5.4-5.9	≤ 2.8 or ≥ 6
Hemoglobin (g/dL)	13-16	11.5-12.9 or 16.1-17	10-11.4 or 17.1-18	≤ 9.9 or ≥ 18.1
WCC (x10^12^/L)	4-10	10.1-20 or 3.1-3.9	≥ 20.1 or ≤ 3	
Electrocardiogram	Normal		AF (60-90)	Any other change
Operative score				
Variable	1 point	2 points	4 points	8 points
Operative magnitude	Minor	Intermediate	Major	Major+
No. of operatrions within 30 days	1		2	>2
Blood loss per operation (mL)	< 100	101-500	501-999	>1000
Peritoneal contamination	No	Serious	Local pus	Free bowel content, pus, or blood
Presence of malignancy	No	Primary cancer only	Node metastases	Distant metastases
Timing of operation	Elective		Emergency resuscitation possible, operation < 24h	Emergency immediate operation<2h
